# Case Report: Abscopal effect and long-term survival in a PD-L1 negative NSCLC patient treated with radiotherapy and immuno-chemotherapy

**DOI:** 10.3389/fimmu.2025.1613974

**Published:** 2025-08-22

**Authors:** Qiang Wen, Weiqi Wang, Ke Zhang, Chunguo Pan, Zhihua Liu, Lei Wang

**Affiliations:** ^1^ Department of Radiation Oncology, Jiangxi Cancer Hospital and Institute, Jiangxi Clinical Research Center for Cancer, The Second Affiliated Hospital of Nanchang Medical College, Nanchang, Jiangxi, China; ^2^ Department of Oncology, Nanchang University, Jiangxi Cancer Hospital and Institute, Nanchang, Jiangxi, China; ^3^ Department of Pathology, Jiangxi Cancer Hospital and Institute, Jiangxi Clinical Research Center for Cancer, The Second Affiliated Hospital of Nanchang Medical College, Nanchang, Jiangxi, China

**Keywords:** NSCLC, brain metastases, abscopal effect, PD-L1 negative, immuno-chemotherapy

## Abstract

We present a case of a 68-year-old male with advanced non-small cell lung cancer (NSCLC), PD-L1 negative and driver gene negative, who exhibited a significant abscopal effect following radiotherapy combined with systemic immunotherapy (sintilizumab) and chemotherapy. The patient achieved complete remission (CR) of intracranial metastases without cranial irradiation, suggesting a systemic immune response triggered by the combination of radiotherapy and immunotherapy. This case highlights the potential of radiotherapy combined with immuno-chemotherapy to induce abscopal effects, even in PD-L1 negative patients, and underscores the importance of further investigation into this therapeutic strategy. This case challenges traditional paradigms in NSCLC management and aligns with emerging theragnostic approaches that integrate localized treatment with systemic immune modulation.

## Introduction

The management of brain metastases in NSCLC traditionally relies on cranial irradiation (1), but emerging evidence supports synergistic effects of radiotherapy (RT) and immunotherapy. The *abscopal effect*, a phenomenon where localized RT induces systemic tumor regression at distant sites, has been increasingly reported in the era of immunotherapy (2). This effect is thought to be mediated by the activation of the immune system, particularly through the release of tumor antigens and subsequent immune response (3, 4). While abscopal effects are rare, they have been observed in various cancers, including NSCLC (5), especially when radiotherapy is combined with immune checkpoint inhibitors. Recent genomic studies highlight that homologous recombination deficiency (HRD) may enhance immunogenicity in driver-negative NSCLC (6), while PD-L1 negativity typically correlates with reduced immunotherapy response. Here, we report a PD-L1 negative NSCLC case achieving rapid intracranial remission through abscopal effects.

## Case presentation

In August 2020, a 68-year-old male patient presented with a right lower lobe pulmonary nodule. Following guidelines at the time, he underwent surgical resection of the primary tumor. The postoperative pathological diagnosis was stage IA (pT1N0M0) right lower lobe adenocarcinoma. No adjuvant therapy was administered. RNA sequencing testing revealed no detectable alterations in the tested driver genes (EGFR, ALK, ROS1, RET, KRAS, BRAF, MET, HER2, and NTRK were all negative). Due to limitations in economic resources and access to advanced clinical testing, extended molecular profiling—including Tumor Mutational Burden (TMB), Microsatellite Instability-High (MSI-H), or Mismatch Repair Deficiency (dMMR)—could not be performed. However, immunohistochemistry (IHC) confirmed negative PD-L1 expression (**Supplementary Figure 1**).

**Figure 1:**
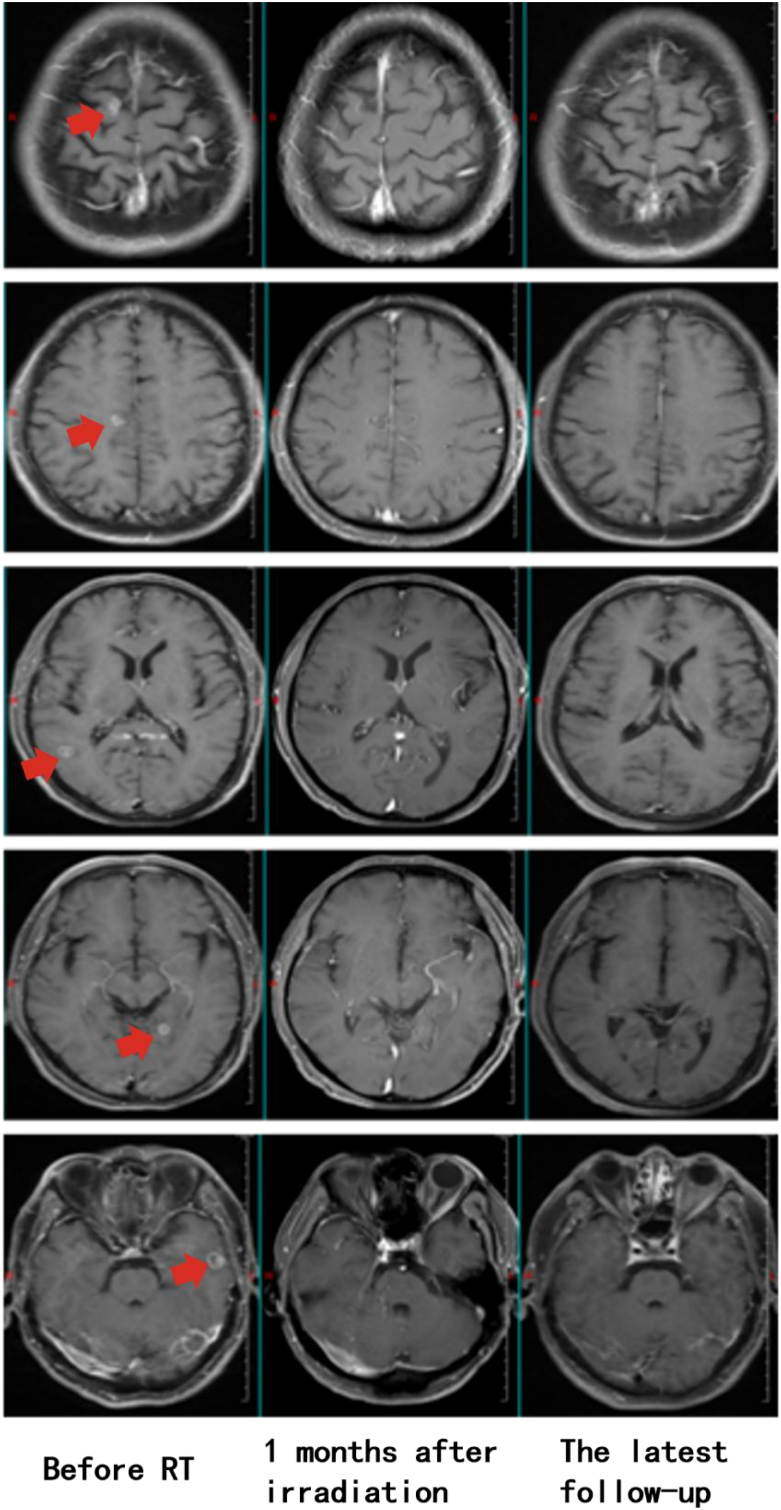
Longitudinal assessment of brain metastasis status following radiotherapy. Pre-treatment imaging (February 7, 2022) confirmed the presence of multiple intracranial metastases prior to radiotherapy for the left iliac bone lesion (Left). Post-therapeutic evaluation at one-month follow-up (March 12, 2022) demonstrated complete radiological response, with no detectable metastatic lesions (Middle). Sustained complete remission was maintained throughout the entire follow-up period, with no evidence of disease recurrence on final imaging studies (January 23, 2025, 35 months post-treatment) (Right).

In February 2022, the patient developed left iliac bone metastasis and multiple intracranial metastases (**Figure 1**), with no new lesions detected at other sites. Given the patient’s left iliac bone pain but absence of central nervous system symptoms, we employed intensity-modulated radiation therapy (IMRT) targeting only the left iliac bone metastatic lesion with a total dose of 36 Gy in 12 fractions to alleviate pain symptoms. (**Supplementary Figure 2**). During the treatment of RT, the patient received one cycle of pemetrexed disodium(500mg/m^2^), cisplatin(75mg/m^2^), and sintilizumab (200mg,an anti-PD-1 antibody). Notably, follow-up MRI revealed complete remission (CR) of multiple brain metastases (**Figure 1**), despite the absence of cranial irradiation one month later.

**Figure 2:**
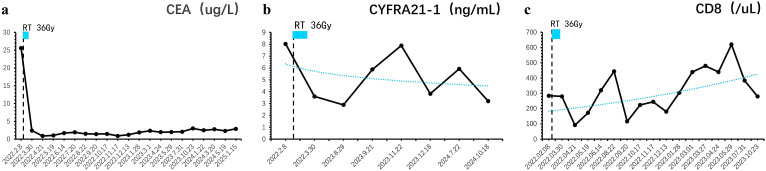
Longitudinal monitoring of tumor markers and immune response dynamics. **(a)** Carcinoembryonic antigen (CEA) levels exhibited a progressive decline post-treatment, eventually stabilizing at baseline values. **(b)** Similarly, cytokeratin 19 fragment (CYFRA 21-1) concentrations demonstrated a sustained reduction, reaching undetectable or minimal levels during follow-up. **(c)** Peripheral immunophenotyping revealed persistently elevated CD8+ T-cell counts, indicative of a robust and durable antitumor immune response.

Subsequently, the patient underwent two cycles of systemic treatment with pemetrexed disodium (500mg/m^2^), cisplatin (75mg/m^2^), and sintilimab (200mg), during which one episode of Grade 3 rash and gastrointestinal reaction occurred. The treatment was then switched to two cycles of carboplatin (AUC=5) combined with pemetrexed disodium (500mg/m^2^) and sintilimab (200mg). Finally, Patients received maintenance treatment with sintilimab (200mg) for 24 months until the last follow-up with or without pemetrexed disodium (500mg/m^2^) alternatively, during which no Grade 2 or higher adverse reactions have occurred. Until the last follow-up, the patient’s lung lesions (**Supplementary Figure 3** **),** bone metastases (**Supplementary Figure 4**), and brain metastases (**Figure 1**) remained stable, with the progression-free survival (PFS) of 35 months and an overall survival (OS) exceeding 40 months (**Table 1**). Prior to each treatment session, peripheral blood tumor markers—including carcinoembryonic antigen (CEA) and cytokeratin 19 fragment (CYFRA 21-1)-were routinely monitored. During the post-treatment surveillance phase after completing the two-year therapeutic regimen, assessment frequency was reduced to quarterly intervals. Results demonstrated progressive decline of these biomarkers, ultimately stabilizing at low levels (**Figure 2**). Concurrently, peripheral CD8+ T-cell counts were evaluated at identical time points using flow cytometry (BD FACSCanto II) with CD3+/CD8+ antibodies, with data indicating persistently elevated levels throughout the observation period (**Figure 2**). Next-generation sequencing of archival tumor tissue identified a *TP53* mutation (VAF 4.8%).The sustained remission of both irradiated and non-irradiated lesions, particularly the brain metastases, underscores the potential of this combined approach to achieve durable disease control in advanced NSCLC, even in PD-L1 negative patients.

**Table 1:** The evolution of lesion status at key timepoints.

Timepoint	Brain Metastases (mm)	Lliac Bone Metastasis (status)	New Lesions	Overall Response
Baseline (February 2022)	46.1	Present	NO	NA
post-radiotherapy (March 2022)	0	Improved	NO	PR
Last follow-up (January 2025)	0	Improved	NO	PR

NA, Not Applicable; PR, Partial Response.

This table documents the longitudinal changes in tumor burden, with target lesions (brain metastases) quantified by the sum of the longest diameters (SLD, mm) of all measurable lesions, non-target lesions (iliac bone metastasis) described by their presence status, and overall response assessed per RECIST 1.1 criteria. Timepoints correspond to: baseline (pre-treatment), post-radiotherapy evaluation, and final follow-up.

## Discussion and conclusion

This case report describes a 68-year-old male with advanced, PD-L1 negative, driver gene-negative NSCLC who achieved complete remission of intracranial metastases after a single cycle of combined radiotherapy, immunotherapy (sintilizumab), and chemotherapy, without cranial irradiation. The treatment, which included IMRT to a left iliac bone metastasis, triggered a systemic immune response, leading to durable disease control and a PFS of 35 months. This notable outcome highlights the potential of combining radiotherapy with immuno-chemotherapy to induce abscopal effects, even in traditionally less responsive PD-L1 negative patients.

The case underscores the importance of multimodal approaches in achieving long-term survival and challenges current paradigms in the management of advanced NSCLC. While the abscopal effect—where localized RT induces systemic tumor regression—has been reported in NSCLC (7–10), the speed of intracranial response in this case is exceptional. The likely mechanism involves RT-induced immunogenic cell death, releasing tumor antigens and damage-associated molecular patterns (DAMPs) that activate dendritic cells and prime tumor-specific T cells (11). The addition of sintilizumab, a PD-1 inhibitor, further amplified this immune response by reversing T cell exhaustion, enabling systemic tumor control, including in the brain (12). Additionally, the patient’s *TP53* mutation, which is associated with increased tumor mutational burden and immunogenicity, may have contributed to the robust abscopal effect by enhancing the presentation of neoantigens and promoting a stronger immune response post-radiotherapy (13).Although the rapid intracranial response observed in this case strongly suggests an abscopal effect, we acknowledge that systemic immunotherapy (sintilimab) and chemotherapy may have contributed to the control of brain metastases. Previous studies indicate that PD-1 inhibitors can cross the blood-brain barrier and exert effects on brain metastases (14, 15). Furthermore, pemetrexed, when combined with platinum-based chemotherapy, has also been reported to exhibit limited intracranial activity (16). However, the observation of a complete response within one month following radiotherapy and just one cycle of systemic therapy is more consistent with the characteristics of a radiation-induced abscopal effect—as the typical response time for systemic therapy alone is usually longer. This case highlights the synergistic potential of radiotherapy combined with immunochemotherapy in achieving rapid and durable systemic responses, even in challenging cases such as advanced non-small cell lung cancer with brain metastases.

The second highlight of this case is the remarkable PFS of 35 months, achieved through maintenance therapy with pemetrexed and sintilimab following initial treatment. The sustained high levels of CD8+ T cells in the patient’s peripheral blood, coupled with low tumor burden, likely contributed to this durable response. RT-induced immunogenic cell death and sintilizumab’s blockade of PD-1/PD-L1 signaling may have synergistically maintained CD8+ T cell activation, preventing T cell exhaustion and promoting continuous anti-tumor immunity (10, 17–19). Additionally, it is worth noting that pemetrexed may enhance immune efficacy through a triple mechanism: (1) up-regulating PD-L1 expression in tumor cells (20); (2) Reduce Treg cell infiltration (21, 22); (3) enhance the sensitivity of tumor cells to T cell killing (23). These mechanisms may work synergistically with radiotherapy and immunotherapy. Previous studies have shown that high peripheral CD8+ T cell levels correlate with improved survival in NSCLC patients receiving immunotherapy (24, 25), as these cells play a critical role in tumor cell recognition and elimination. This case underscores the importance of combining radiotherapy, immunotherapy, and chemotherapy to sustain immune activation and achieve long-term disease control, even in advanced NSCLC with high-risk features.

This case represents a rare and remarkable example of rapid intracranial remission and long-term survival in a PD-L1 negative, driver gene-negative NSCLC patient treated with a combination of RT and immuno-chemotherapy. The rapid speed of intracranial response and sustained disease control highlight the potential of multimodal therapy to induce systemic immune activation and achieve durable outcomes in traditionally challenging cases. The integration of localized RT with systemic immuno-chemotherapy exemplifies a theragnostic approach (26), where molecular imaging (e.g., CXCR4-targeted PET) could non-invasively monitor immune activation during combined modality therapy. Such approaches may optimize RT/immunotherapy sequencing in PD-L1 negative NSCLC. Future studies could utilize patient-derived organoids (PDOs) to model such abscopal responses. As demonstrated in NSCLC PDOs (27), these models can recapitulate tumor-immune interactions and predict combinatorial therapy efficacy, potentially identifying biomarkers for patient selection.

## Data Availability

The original contributions presented in the study are included in the article/**Supplementary Material**. Further inquiries can be directed to the corresponding author.
