# Supplementary material for: Case Report: Abscopal effect and long-term survival in a PD-L1 negative NSCLC patient treated with radiotherapy and immuno-chemotherapy
Source: Front Immunol. 2025 Aug 22;16:1613974. doi: 10.3389/fimmu.2025.1613974 (PMC12411182; doi:10.3389/fimmu.2025.1613974)
Supplement: Supplementary file 1 [file DataSheet1.docx]

### **Case Report: Abscopal Effect and Long-Term Survival in a PD-L1 Negative NSCLC Patient Treated with Radiotherapy and Immuno-Chemotherapy**

Qiang Wen^1†^, Weiqi Wang^2†^, Ke Zhang^3^, Chunguo Pan^1^, Zhihua Liu^1^, Lei Wang^1*^

^1^Department of Radiation Oncology, Jiangxi Cancer Hospital & Institute, Jiangxi Clinical Research Center for Cancer, The Second Affiliated Hospital of Nanchang Medical College, Nanchang 330029, Jiangxi Province, China

^2^Department of Oncology, Nanchang University, Nanchang,Jiangxi, China

^3^Department of Pathology, Jiangxi Cancer Hospital & Institute, Jiangxi Clinical Research Center for Cancer, The Second Affiliated Hospital of Nanchang Medical College, Nanchang 330029, Jiangxi Province, China

† contributed equally to this works

*Correspondence:

Lei Wang, Department of Radiation Oncology, Jiangxi Clinical Research Center for Cancer, Jiangxi Cancer Hospital, The Second Affiliated Hospital of Nanchang Medical College, Nanchang, Jiangxi, China. E-mail: wangleiy001@126.com


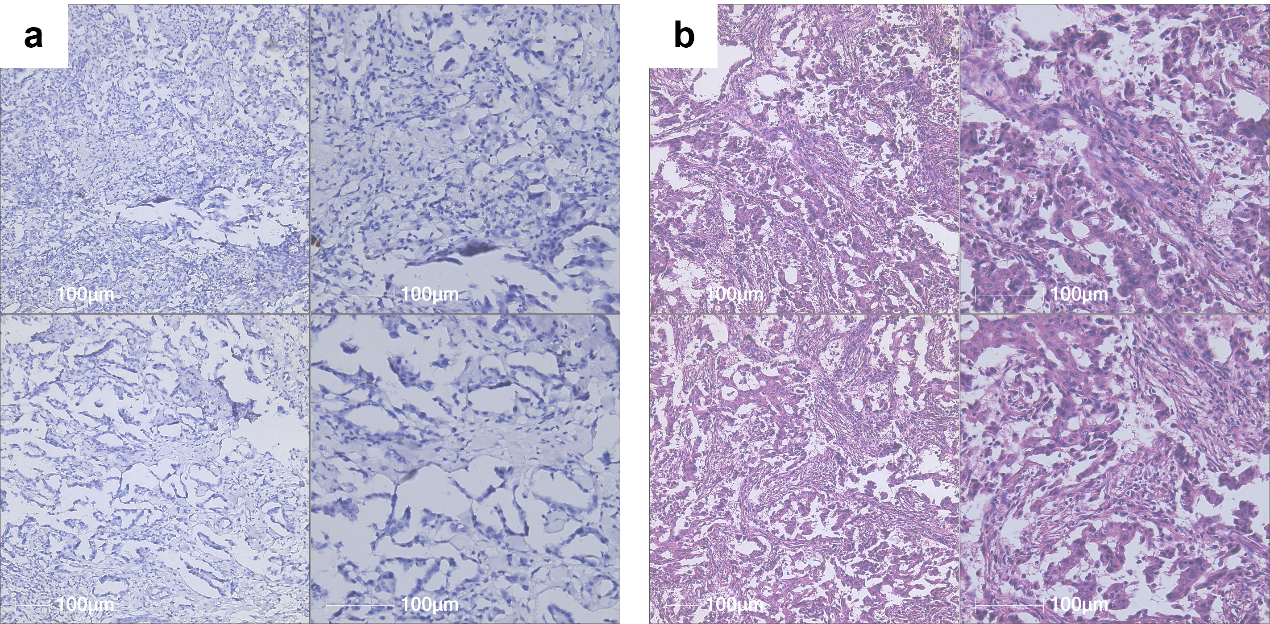


Supplementary Fig 1 Histological findings of the tumor in the right lower lung lobe. Scale bar, 100 μm.(a) unstained;(b) H&E-stained.


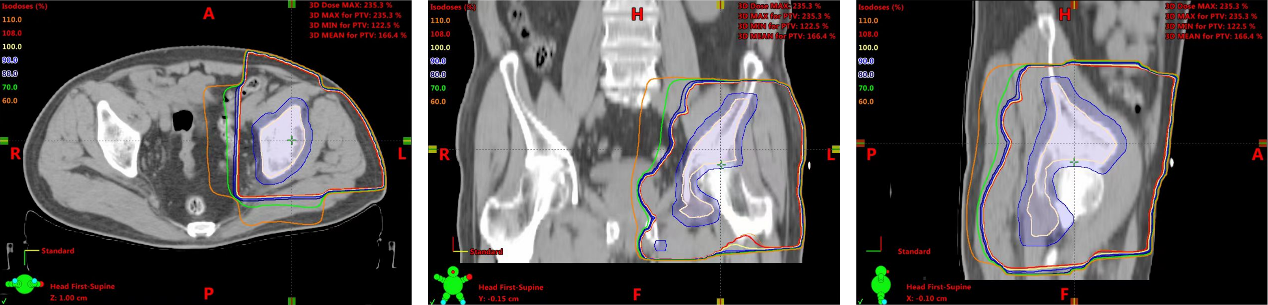


Supplementary Fig 2 Intensity-Modulated Radiation Therapy (IMRT) was applied, with axial (left), coronal (middle), and sagittal (right) images selected. The left iliac bone metastasis served as the Clinical Target Volume (CTV). A 0.3 cm margin was expanded from the CTV to form the planning target volume (implied by context). The prescription dose, defined by the 100% isodose line, totaled 36 Gy delivered in 12 fractions. Isodose lines represent: 36 Gy (blue, total dose), 25 Gy (green), and 22 Gy (orange).


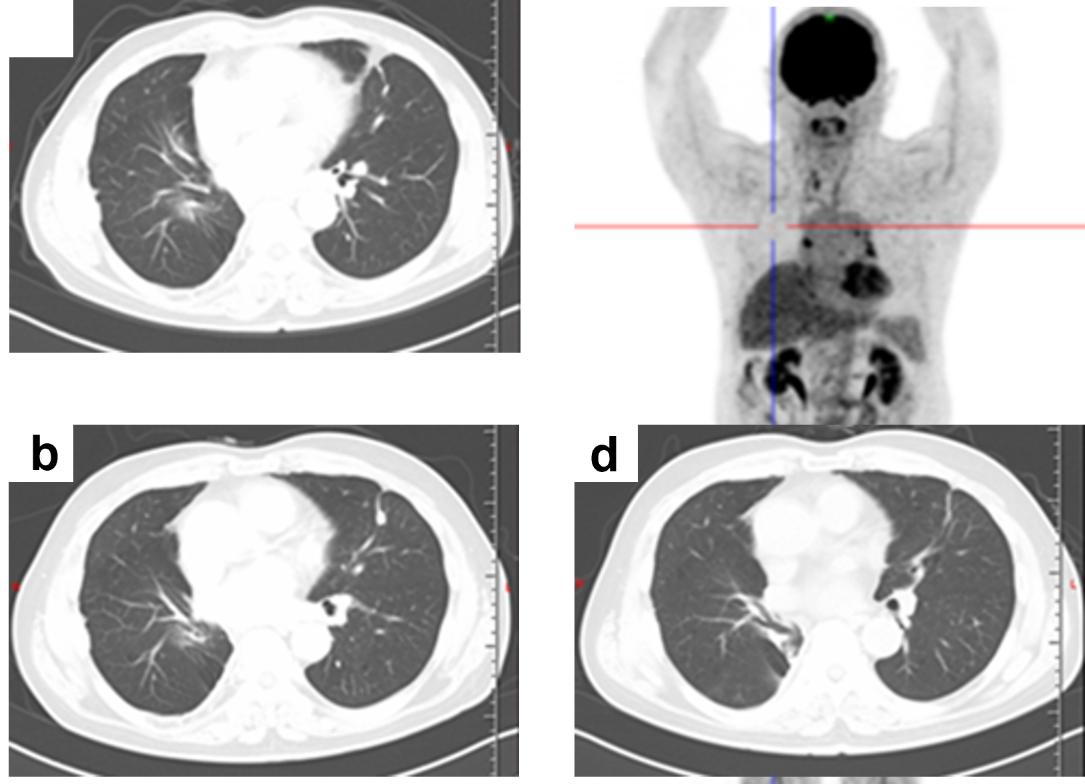


Supplementary Fig 3 The computed tomography scan of the chest revealed no recurrence of pulmonary lesions:(a) Before RT;(b) First postoperative follow-up;(d) The latest follow-up.

PET-CT revealed:(c) no recurrence of pulmonary lesions was observed.


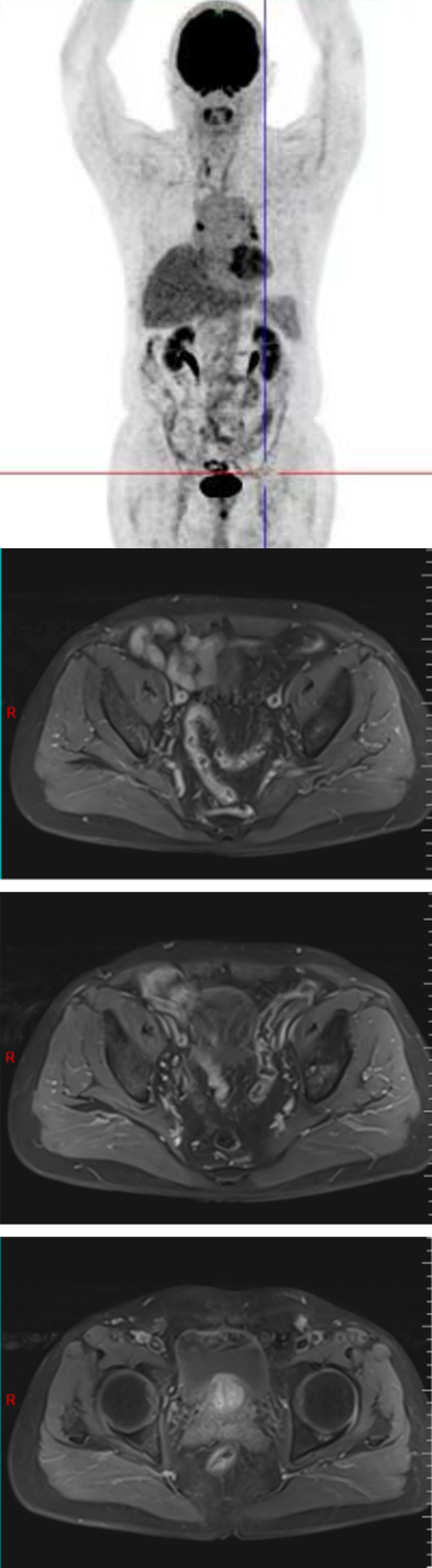


Supplementary Fig 4 The latest PET-CT：Cerebral metastases: no evidence of metastatic lesions. Iliac bone metastasis: metabolic regression noted.
